# Ancestry-informative regulatory variants at KCNB1 modulate adipogenesis and body mass index

**DOI:** 10.3389/fendo.2026.1805824

**Published:** 2026-05-07

**Authors:** Samantha Kuwada Teixeira, Caroline Pancera Laurindo, Fábio Takeo Sato, Luiz Fernando Goda Zuleta, Nubia E. Duarte, Ana Vitória V. Jensen, Samantha L. G. Paço, Alexandre C. Pereira, Noboru Sakabe, Marcelo Nobrega, Jose Eduardo Krieger

**Affiliations:** 1 Instituto do Coração, Hospital das Clínicas HCFMUSP, Faculdade de Medicina, Universidade de São Paulo, Sao Paulo, SP, Brazil; 2 Department of Human Genetics, University of Chicago, Chicago, IL, United States

**Keywords:** adipogenesis, admixed population, ancestry-specific variants, BMI, body composition, KCNB1, obesity

## Abstract

**Introduction:**

Obesity and related metabolic disorders represent a major global health burden, yet their genetic determinants remain incompletely characterized, particularly in non-European populations. We aimed to identify body mass index (BMI)–associated loci in an admixed Brazilian population and to functionally characterize ancestry-enriched variants contributing to obesity risk.

**Methods:**

We conducted a genome-wide association study (GWAS) of BMI in 1,079 admixed Brazilian individuals. Significant and suggestive loci were evaluated using integrative analysis, including epigenomic annotation and chromatin conformation data. Regulatory activity was assessed using allele-specific luciferase reporter assays and electrophoretic mobility shift assays. The functional role of the prioritized gene was examined using pharmacological inhibition in human preadipocytes and genetic deletion in mice.

**Results:**

We identified three BMI-associated loci reaching genome-wide significance (p ≤ 5 × 10^-8^) and 49 additional loci previously implicated in obesity-related traits at suggestive significance. Integrative analyses prioritized a non-coding variant at chromosome 20 (rs149309426), located within an active enhancer that physically interacts with the *KCNB1* promoter during preadipocyte differentiation. The BMI risk allele (C) increased enhancer activity in luciferase assays and showed enhanced transcription factor binding. Pharmacological inhibition of KCNB1 impaired adipocyte differentiation and lipid accumulation in human preadipocytes. Consistently, Kcnb1 knockout mice exhibited reduced fat mass and increased lean mass. The rs149309426 risk allele was rare in European populations but enriched in individuals with African ancestry.

**Discussion:**

Our findings identify KCNB1 as a regulator of adipogenesis and body composition and highlight the importance of studying admixed populations to uncover ancestry-specific genetic mechanisms underlying obesity.

## 1 Introduction

Obesity and its related comorbidities have increased worldwide over the past decades, becoming a major challenge for public health and for the management of metabolic disorders such as type 2 diabetes, dyslipidemia, and cardiovascular disease (1). Body mass index (BMI) remains the most widely used anthropometric measure to define overweight and obesity (2). Although BMI has a limited sensitivity for detecting excess adiposity, it maintains good specificity for obesity and continues to serve as a practical and effective screening tool for large-scale epidemiological and clinical studies (3–6).

Obesity arises from a complex interplay between environmental, behavioral and genetic factors. Heritability estimates of BMI range from 25% to 50%, underscoring the substantial contribution of genetic variation to adiposity and energy balance (7–9). Understanding the genetic architecture of obesity is essential to identify new molecular mechanisms and improve strategies for prevention and treatment.

Over the past two decades, genome-wide association studies (GWAS) have identified hundreds of loci associated with BMI and related traits, providing novel insights into the biology of energy homeostasis, adipocyte differentiation, and appetite regulation (10–14). However, more than 70% of GWAS participants are of European ancestry, resulting in a persistent Eurocentric bias limiting discovery of ancestry-specific variants and transferability of findings to other populations. Genetic diversity strongly influences linkage disequilibrium structure and allele frequencies, meaning that variants common in one population may be rare or absent in another. This imbalance hinders the global applicability of genetic risk prediction and the identification of causal variants, contributing to widening health disparities in genomic medicine (15–17).

Admixed populations, such as those in Brazil, shaped by the admixture of European, African, and Native Americans ancestries (18, 19), offer unique opportunities to overcome these limitations. The combination of distinct ancestral haplotypes within the same genomes enhances fine-mapping resolution, improves power to detect associations, and enables the discovery of ancestry-specific risk alleles (20–23). Yet, admixed cohorts remain underrepresented in human genetics, largely due to challenges in modeling population structure and the lack of large-scale resources from low and middle-income countries (24). Thus, increasing their inclusion is therefore not only a matter of equity but also a scientific imperative to capture the full spectrum of human genetic diversity and its impact on complex traits and disease.

Beyond population diversity, a major challenge in GWAS interpretation is that most associated variants lie within non-coding regions of the genome, suggesting that they affect gene regulation rather than protein structure and function (25, 26). Integrating epigenomic annotations, chromatin interaction data, and gene expression profiles is essential to connect statistical associations with functional mechanisms. This integrative strategy allows the identification of regulatory variants that alter enhancer activity, transcription factor binding, and ultimately gene expression. In adipose tissue, such regulatory variants may alter enhancer activity, thereby modulating adipocyte differentiation and lipid storage, key processes that shape individual susceptibility to obesity and metabolic dysfunction (27).

Here, we conducted a GWAS of BMI in an admixed Brazilian population sample, followed by a multi-layer functional investigation to uncover candidate genes and regulatory mechanisms underlying BMI variation. We identified 52 BMI-associated loci, including three novel loci reaching genome-wide significance. Integrative analysis combining epigenomic and chromatin conformation data revealed regulatory variants affecting *IL10RB* and *KCNB1*, the latter representing a link between ion channel function and adipose biology. Together, our findings expand the genetic landscape of BMI and underscore the value of integrating diverse populations and function genomics to elucidate the inter-individual variability in metabolic risk and obesity susceptibility.

## 2 Results

### 2.1 Genome-wide discovery of BMI-associated loci in an admixed Brazilian population sample

**Figure 1:**
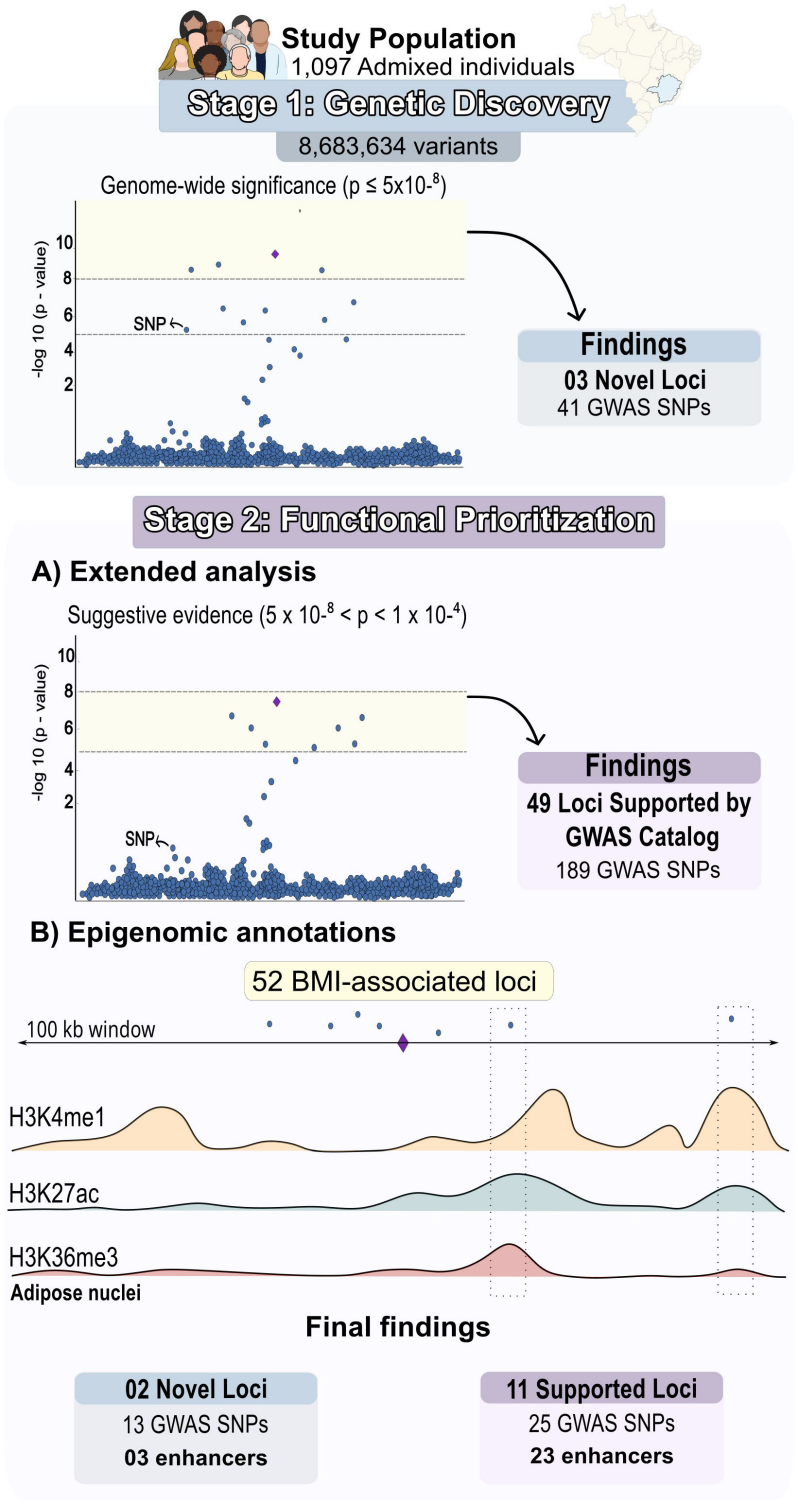
Two-stage analytical workflow with key findings. In Stage I (Genetic Discovery), a BMI GWAS in 1,079 admixed Brazilian individuals identified three novel loci surpassing the genome-wide significance threshold (*p* ≤ 5 × 10^-8^). In Stage II (Functional Prioritization), the analysis was extended beyond these novel loci to include 49 previously reported loci reaching suggestive significance (5 × 10^-8^ < *p* < 1 × 10^-4^), followed by integration with epigenomic annotations from adipose nuclei. This approach identified two novel loci and 11 previously reported loci harboring variants within active or genic enhancer regions, prioritizing candidate regulatory elements for downstream functional analysis.

We performed a GWAS of BMI in 1,079 Brazilian individuals of admixed ancestry (**Supplementary Table 1**). To balance discovery with interpretability in the context of a modest sample size, our analytical strategy was structured in two sequential stages: (i) genetic discovery, aimed at identifying BMI-associated loci using conventional GWAS thresholds, and (ii) functional prioritization, in which a broader set of reproducible associations was evaluated using epigenomic and experimental evidence (**Figure 1**).

In the discovery stage, we tested 8,683,634 variants for association with BMI using a polygenic mixed model accounting for age, sex, age², population structure, and relatedness. Three lead variants at loci on chromosomes 9, 16, and 21 surpassed the conventional genome-wide significance threshold (*p* ≤ 5 × 10^-8^; **Supplementary Figure 1**). Of these, the locus on chromosome 9 remained significant after correction for multiple testing (*p* ≤ 5 × 10^-9^; **Supplementary Table 2**). None of the three loci had been previously reported to be associated with obesity-related traits in the GWAS Catalog.

Across these loci, the BMI-associated alleles showed consistent directionality of effect, with positive β estimates indicating increased BMI among carriers. However, given the limited power inherent to the sample size, we considered genome-wide significant findings as discovery signals requiring additional functional or external support.

### 2.2 Functional prioritization of BMI-associated loci using a polygenic framework

Recognizing the highly polygenic architecture of BMI and evidence that variants below genome-wide significance collectively explain substantial heritability, we extended our analysis to a functional prioritization stage. In this stage, we evaluated variants showing suggestive evidence of association (5 × 10^-8^ < *p* < 1 × 10^-4^), while applying constraints to limit false-positive findings.

Specifically, variants were retained only if they mapped to loci previously associated with obesity-related traits in the GWAS Catalog. To ensure comparability, we applied the same suggestive significance threshold (5 × 10^-8^ < *p* < 1 × 10^-4^) both to our GWAS results and to cataloged associations. This dual-threshold strategy prioritizes loci with independent evidence of association across studies, while avoiding over-reliance on statistical significance alone (**Figure 1**).

Using this approach, we identified 813 loci comprising 3,439 variants associated with BMI at *p* < 1 × 10^-4^ in our GWAS (**Supplementary Table 3**). Of these, 49 loci (189 variants) overlapped with previously reported obesity-related loci in the GWAS Catalog (**Supplementary Table 4**). Together with the three loci reaching genome-wide significance, this yielded a total of 52 BMI-associated loci carried forward for functional integration (**Figure 1**).

### 2.3 Integration of epigenomic annotations identifies regulatory variants in adipose tissue

To assess the functional relevance of BMI-associated variants, we intersected the 52 prioritized loci with epigenomic annotations from adipose nuclei cells generated by the Roadmap Epigenomics Consortium. We focused on variants located within enhancer regions defined by chromatin-state segmentation, as most GWAS signals map to non-coding regulatory elements (**Figure 1**).

**Figure 2:**
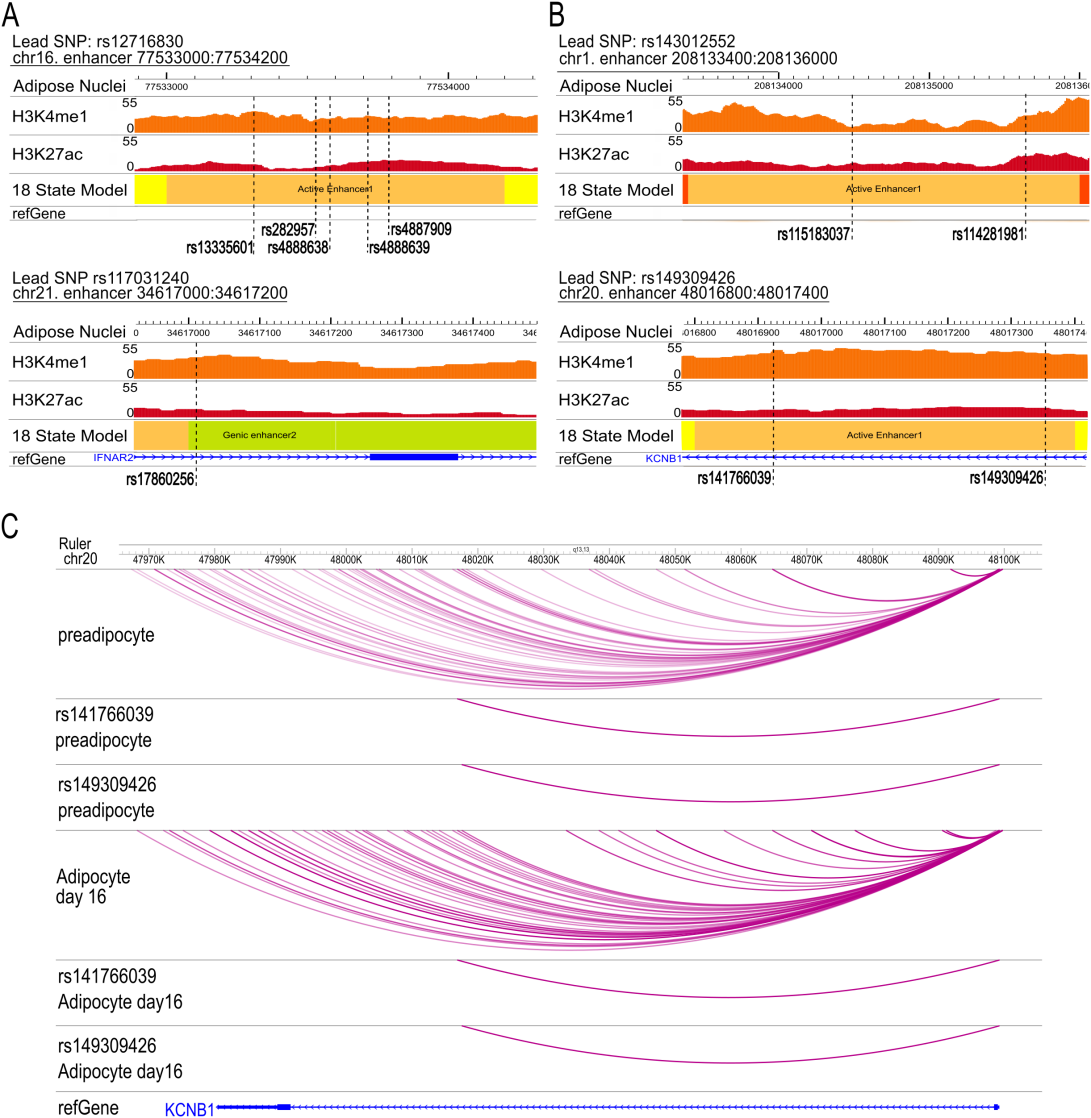
Epigenomic annotation and chromatin interactions at prioritized BMI-associated loci. **(A)** Epigenomic annotation of genome-wide significant BMI-associated loci identified in the discovery stage. Shown are an active enhancer at the chromosome 16 locus harboring five BMI-associated variants marked by H3K4me1 and H3K27ac, and a genic enhancer at the chromosome 21 locus harboring a single BMI-associated variant marked by H3K4me1 and H3K36me3 in adipose nuclei. **(B)** Epigenomic annotation of enhancer regions at BMI-associated loci, during the functional prioritization stage. Active enhancers at the chromosome 1 and chromosome 20 loci harbor two BMI-associated variants each, including rs141766039 and rs149309426 at the chromosome 20 locus. These loci did not reach genome-wide significance but were retained based on independent genetic support across studies and localization within active regulatory elements in adipose tissue. **(C)** Enhancer–promoter interactions linking prioritized BMI-associated variants to *KCNB1*. Promoter capture Hi-C data from human SGBS preadipocytes and differentiated adipocytes demonstrate physical interactions between enhancer regions containing rs141766039 and rs149309426 and the *KCNB1* promoter across multiple stages of adipocyte differentiation, providing a mechanistic link between genetically prioritized regulatory variants and a candidate effector gene. Epigenomic tracks and chromatin interaction maps were visualized using the WashU Epigenome Browser (https://epigenomegateway.wustl.edu/).

Among the three genome-wide significant loci, no enhancer-overlapping variants were identified at the chromosome 9 locus. In contrast, loci on chromosomes 16 and 21 harbored 11 and 2 enhancer-overlapping variants, respectively (**Supplementary Table 5**). Chromatin-state annotation revealed that these enhancers included both active and genic enhancers, marked by combinations of H3K4me1, H3K27ac, and H3K36me3 (**Figure 2A**).

Across the 49 loci prioritized at the suggestive threshold, 11 loci contained 22 enhancer regions harboring 25 BMI-associated variants (**Supplementary Table 6**). Notably, two active enhancers, one at the chromosome 1 locus and one at the chromosome 20 locus, each contained two BMI-associated variants (**Figure 2B**), making them particularly compelling candidates for regulatory function.

### 2.4 Chromatin interaction data nominate *KCNB1* as a candidate effector gene

To link enhancer-associated variants to their target genes, we integrated promoter capture Hi-C data generated in human SGBS preadipocytes (Simpson–Golabi–Behmel syndrome–derived preadipocytes, 12) and adipocytes across multiple stages of differentiation. Among the genome-wide significant loci, only the chromosome 21 enhancer harboring rs17860256 showed physical interaction with a gene promoter, specifically *IL10RB*, at day 8 of adipocyte differentiation (**Supplementary Table 5**).

Across the suggestive loci, nine enhancer-associated variants showed reproducible interactions with gene promoters (**Supplementary Table 6**). Of particular interest, the active enhancer at the chromosome 20 locus contained two BMI-associated variants, rs141766039 and rs149309426, both of which interacted with the *KCNB1* promoter in preadipocytes and at later stages of differentiation (**Figure 2C**). RNA-seq data indicated that *KCNB1* expression increased during adipocyte differentiation before declining at terminal stages, consistent with a role in adipogenic programming (**Supplementary Figure 2**).

To further prioritize candidate causal variants at this locus, we performed statistical fine-mapping using the SuSiE framework (28). This analysis identified credible sets with distributed posterior inclusion probabilities, consistent with local linkage disequilibrium structure. Among enhancer-associated variants, rs7594699 was prioritized with high probability (PIP = 0.99); however, this variant maps to a weak enhancer and showed no evidence of interaction with gene promoters in our chromatin interaction data. In contrast, rs141766039 and rs149309426 were not assigned high PIPs, likely reflecting their strong linkage disequilibrium and the resulting sharing of posterior probability across correlated variants (**Supplementary Table 7**).

Importantly, the BMI-increasing alleles at rs141766039 and rs149309426 were rare in European populations but occurred at higher frequencies in African ancestry populations and at appreciable frequencies (>1%) in the Brazilian cohort (**Supplementary Table 8**). The higher frequency of the rs149309426 risk allele in individuals with partial African ancestry suggests that local ancestry may modulate detectability at this locus, an effect that would be diluted in homogeneous European cohorts. This population distribution, together with convergent epigenomic and chromatin interaction evidence, motivated prioritization of the chromosome 20 locus for allele-specific functional validation.

### 2.5 Allele-specific enhancer activity at rs149309426

To directly test whether the prioritized variants modulate enhancer activity, we performed luciferase reporter assays using 500 bp enhancer fragments encompassing rs141766039 or rs149309426, carrying either the BMI risk or non-risk allele. Assays were conducted in human preadipocytes and adipocytes under basal and inflammatory conditions.

**Figure 3:**
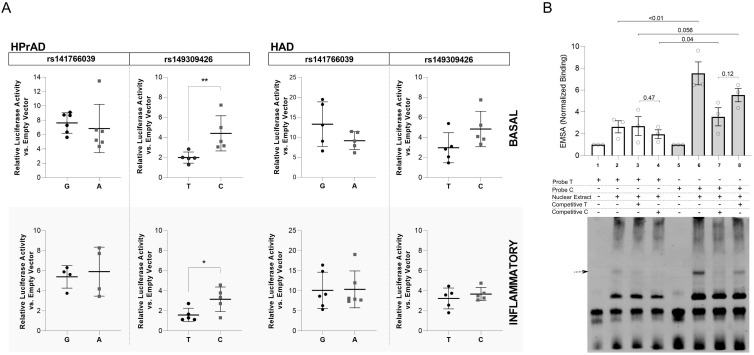
Allele-dependent enhancer activity and transcription factor binding at rs149309426. **(A)** Luciferase reporter assays in human preadipocytes and adipocytes showing increased enhancer activity associated with the rs149309426 C allele, which is directionally concordant with increased BMI in the GWAS. **(B)** Electrophoretic mobility shift assays demonstrating stronger protein binding to the C allele compared to the T reference allele, consistent with enhanced regulatory activity. These experiments provide functional validation of a variant prioritized through the GWAS-to-function framework.

The rs149309426 C allele, which was associated with higher BMI in the GWAS, significantly increased enhancer activity in preadipocytes under both basal and inflammatory conditions, whereas no allele-dependent effect was observed in mature adipocytes (**Figure 3A**). In contrast, rs141766039 showed no detectable effect on enhancer activity in either cell types. Thus, genetic effect directionality at rs149309426 was concordant with increased regulatory activity in an adipogenic context.

Electrophoretic mobility shift assays further demonstrated stronger protein binding to the rs149309426 C allele compared to the reference allele, consistent with enhanced transcription factor affinity (**Figure 3B**). Genotyping of rs149309426 in the Brazilian cohort using an independent assay confirmed the association with BMI (p = 0.046), validating the imputed genotype. In silico motif analysis predicted that the variant alters binding motifs for SIN3A and PAX5 transcription factors (**Supplementary Table 9**), indicating potential allele-specific regulatory activity. Notably the predicted allele-specific binding differences are consistent with the differential band patterns observed in EMSA experiments, supporting the presence of sequence-specific transcription factor binding at this locus.

### 2.6 Functional evidence supports a role for *KCNB1* in adipogenesis and body composition

**Figure 4:**
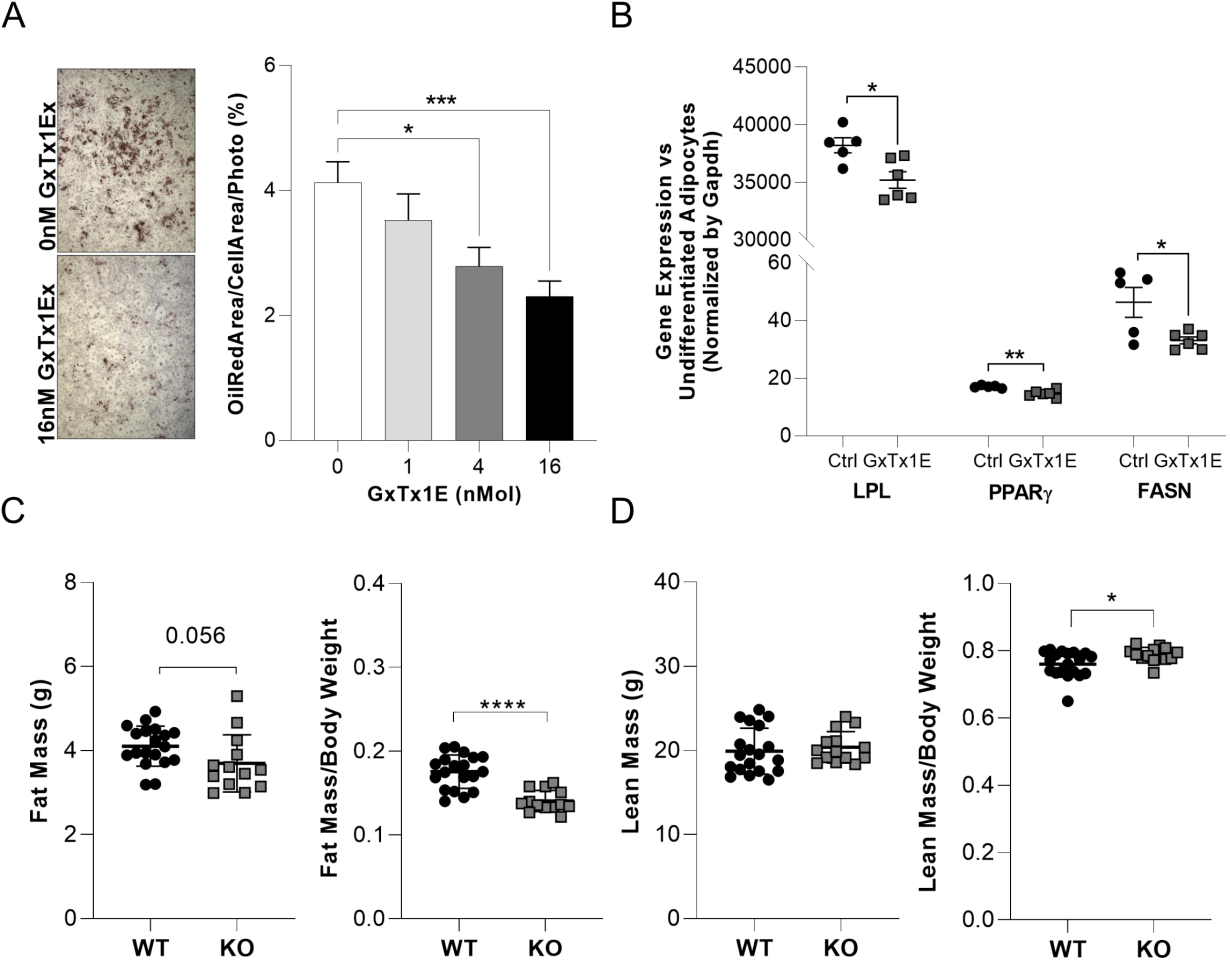
Functional validation of KCNB1 in adipogenesis and body composition. **(A)** Representative Oil Red O staining of human preadipocytes treated with GxTx1E (KCNB1 inhibitor, from 0 to 16nM) and quantification of lipid accumulation across increasing inhibitor concentrations. **(B)** Reduced expression of adipogenic markers (*PPARγ, LPL, FASN*), as assessed by qRT-PCR, following KCNB1 inhibition. **(C)** Body composition analysis of *Kcnb1* knockout (KO) and wild-type mice from the International Mouse Phenotyping Consortium, showing reduced fat mass and fat mass–to–body weight ratio in KO animals and **(D)** increased lean mass and lean mass–to–body weight ratio Kcnb1 knockout. *p≤ 0.03; **p=0.002; ***p=0.0007; ****p=0.0002.

Given the allele-dependent regulatory effects at the *KCNB1* locus, we next evaluated the functional role of *KCNB1* in adipogenesis. Pharmacological inhibition of *KCNB1* during differentiation of human preadipocytes resulted in a dose-dependent reduction in lipid accumulation, accompanied by decreased expression of key adipogenic markers (*PPARγ*, *LPL*, and *FASN*) (**Figures 4A, B**).

To assess *in vivo* relevance, we analyzed publicly available phenotypic data from *Kcnb1* knockout mice generated by the International Mouse Phenotyping Consortium (29, 30). Loss of *Kcnb1* was associated with reduced fat mass and increased lean mass normalized to body weight (**Figures 4C, D**), supporting a conserved role for *KCNB1* in adipose development and systemic energy balance.

Together, these results indicate that the BMI-associated rs149309426 C allele increases enhancer activity at the *KCNB1* locus, likely promoting higher *KCNB1* expression during adipocyte differentiation, which in turn contributes to increased adiposity.

## 3 Discussion

**Figure 5:**
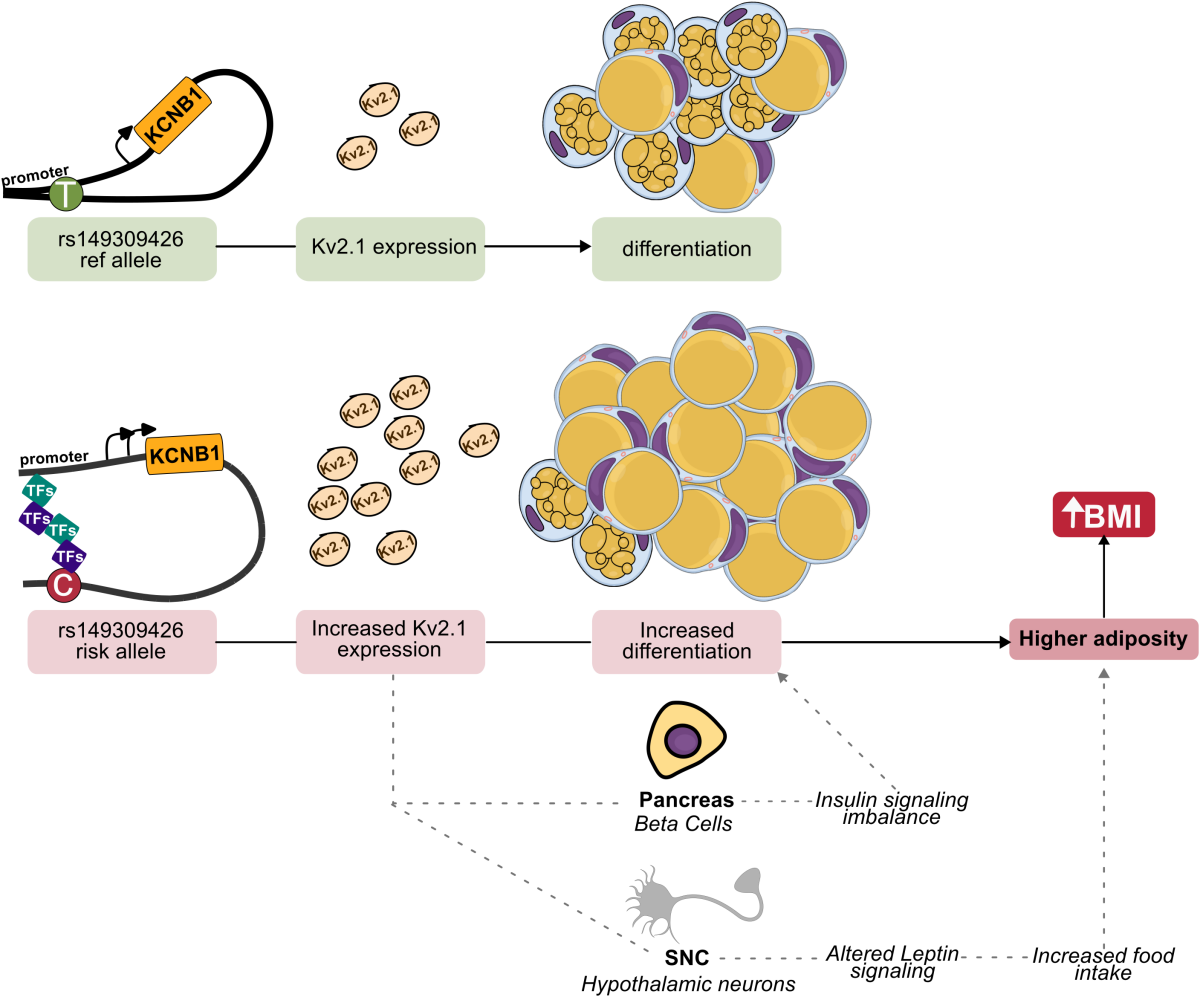
Proposed model linking regulatory variation at the KCNB1 locus to adipogenesis and BMI. The BMI-associated rs149309426 C allele enhances enhancer activity and transcription factor binding, promoting increased *KCNB1* expression during early adipocyte differentiation. Elevated KCNB1 activity promotes adipocyte differentiation and fat mass accumulation, contributing to increased BMI. Dashed arrows indicate regulatory effects of KCNB1 in pancreatic β cells and central nervous system (hypothalamic neurons) that have been reported in the literature but were not directly tested in this study, reflecting an integration of statistical discovery, functional prioritization, and experimental validation.

In this study, we integrated ancestry-aware genome-wide association analyses with functional genomics to identify regulatory variation contributing to body mass index. Using an admixed Brazilian cohort, we uncovered both genome-wide significant and reproducible suggestive BMI-associated loci and, through systematic functional prioritization, KCNB1 was identified as a biologically relevant effector gene. Our results link non-coding genetic variation at the chromosome 20 locus to adipogenesis and body composition, providing mechanistic insight into the genetic architecture of obesity (**Figure 5**).

### 3.1 GWAS power, polygenicity, and ancestry-aware discovery

Genome-wide association studies have substantially advanced our understanding of complex traits by enabling the detection of common variants with small individual effects. However, the highly polygenic nature of BMI, together with the limited power of modestly sized cohorts, constrains discovery when analyses rely exclusively on stringent genome-wide significance thresholds. Variants showing suggestive evidence of association can nonetheless capture meaningful components of heritability and are often enriched for true biological signals. Accordingly, we adopted a two-stage framework that separated statistical discovery from functional prioritization, allowing downstream analyses to focus on variants supported by both genetic evidence and regulatory plausibility.

To date, more than 70% of GWAS participants are of European ancestry, creating a persistent Eurocentric bias that constrains the generalizability, equity, and translational impact of genetic findings (15, 31). This imbalance limits the detection of ancestry-specific variants and can lead to population-dependent effect-size estimates and reduced portability of genetic associations across diverse groups. Although initiatives to broaden representation are expanding, admixed populations, particularly from Latin America and Africa, remain markedly underrepresented due to structural, funding, and methodological challenges.

Admixed populations offer distinct advantages for genetic discovery because differences in allele frequencies and linkage disequilibrium patterns inherited from multiple ancestral sources can increase power for fine-mapping and reveal regulatory variants that are rare or absent in European populations. Simulations by Lin et al. further demonstrate that causal variants under differential selective pressures across ancestries may reach intermediate allele frequencies in admixed populations, enhancing their detectability in GWAS (20). In this study, the identification of rs149309426, a BMI-associated variant rarely found in Europeans but more frequent in individuals with African ancestry, illustrates how ancestry-aware GWAS can uncover biologically meaningful signals that would likely remain undetected in Eurocentric studies.

We further interrogated GWAS results from an independent African-ancestry cohort of the VA Million Veteran Program; however, rs149309426 did not show evidence of association with BMI in this dataset (p = 0.643 and p = 0.95 using harmonized ancestry and race/ethnicity [HARE] and genetically informed ancestry [GIA], respectively) (32). While this lack of replication may suggest population-specific effects, its interpretation requires consideration of the unique genetic architecture of admixed Brazilian populations. Recent evidence shows that African ancestry segments in Brazilian genomes are highly diverse, deriving from multiple subcontinental regions—including West, East, South, and North Africa—resulting in mosaic genomes shaped by historical admixture among populations that may not have interacted prior to the transatlantic diaspora (18). Such complex ancestry mosaics can generate distinct local haplotypic and regulatory contexts, potentially modulating genetic effects and contributing to differences in detectability across populations.

Together, these findings underscore that inclusion of admixed populations is not only a matter of equity but a scientific necessity for resolving the genetic architecture of complex traits such as obesity.

### 3.2 Variant prioritization and functional integration

Given the modest sample size and ongoing debate surrounding the “missing heritability” problem (33), we adopted a polygenic framework for variant prioritization. Multiple studies have shown that variants below the genome-wide significance threshold collectively explain a substantial fraction of heritability and should not be excluded from downstream analyses (34–36). Suggestive associations (5 × 10^-8^ < *p* < 1 × 10^-4^) are enriched for true signals and can improve polygenic prediction (37). Consistent with the polygenic nature of BMI, we therefore expanded our analyses beyond the conventional significance threshold.

To limit false positives, we restricted analyses to loci previously associated with obesity-related traits in the GWAS Catalog, anchoring our results in established biological context. To avoid redundancy with extensively characterized regions, we further prioritized variants reported only at suggestive significance levels rather than well-established genome-wide significant loci. This strategy enabled us to focus on comparatively understudied regulatory variants with plausible functional relevance.

### 3.3 Regulatory mechanisms at the *KCNB1* locus

Applying this framework, we prioritized rs149309426, a non-coding variant located within an active enhancer that physically interacts with the *KCNB1* promoter during adipocyte differentiation. Allele-specific luciferase reporter assays and electrophoretic mobility shift assays demonstrated that the BMI-increasing allele enhances regulatory activity and transcription factor binding, establishing a direct link between genetic association and gene regulation.

Functional perturbation experiments further supported a role for *KCNB1* in adipogenesis. Its pharmacological inhibition impaired adipocyte differentiation and lipid accumulation in human preadipocytes, while *Kcnb1* knockout mice exhibited reduced fat mass and increased lean mass normalized to body weight. Together, these findings identify *KCNB1* as a regulator of adipose development and body composition.

### 3.4 Additional effector genes and locus prioritization

Our integrative analyses also highlighted *IL10RB* as a candidate effector gene at the novel chromosome 21 locus. Although *IL10RB* expression did not display a clear trajectory during adipocyte differentiation, chromatin interaction data revealed regulatory contact between a BMI-associated variant and the *IL10RB* promoter. *IL10RB* encodes the β-chain of the interleukin-10 receptor, which has been implicated in energy expenditure, adipose browning, and inflammatory responses (38–41). Prior associations with fat percentage (42) and proteomic links to body fat mass (43) further support its relevance. Consistent with our Hi-C data, rs17860256 acts as an eQTL for *IL10RB* in fibroblasts according to GTEx (44).

While these findings validate our integrative strategy, *IL10RB* was not prioritized for downstream functional validation in this study due to its well-established role in metabolic regulation, and instead serves as contextual support for the locus. We therefore prioritized downstream functional validation of the chromosome 20 locus, previously associated with energy expenditure at suggestive significance in Hispanic children (rs6063399; *p* = 8 × 10^-6^) (45).

### 3.5 Biological roles of *KCNB1* across tissues

*KCNB1* encodes the voltage-gated potassium channel Kv2.1, which regulates membrane excitability in multiple tissues, including adipose tissue (46, 47), skeletal and cardiac muscle, brain (48), and pancreas (49). In pancreatic β-cells, Kv2.1 regulates glucose-induced action potentials and insulin secretion; loss of *Kcnb1* enhances glucose-stimulated insulin release. However, Kv2.1 also forms non-conducting membrane clusters that promote insulin exocytosis independently of ion flux (50–52). In humans, genetic variation in *KCNB1* has been associated with insulin resistance, dyslipidemia, and central adiposity (53, 54), suggesting broader metabolic roles.

In adipose tissue, insulin modulates the activity of potassium channels in adipocytes, increasing K^+^ currents across the plasma membrane; this effect becomes more pronounced during adipocyte differentiation and correlates with enhanced adipogenesis (55). Consistent with this, *KCNB1* expression increases during terminal adipocyte differentiation (56). These observations align with our findings that pharmacological inhibition of *KCNB1* impairs adipocyte differentiation and lipid accumulation, and that *Kcnb1* knockout mice display reduced fat mass and increased lean mass normalized to body weight. Conversely, the rs149309426 risk allele increased enhancer activity *in vitro* and was associated with higher BMI in our cohort, supporting the interpretation that upregulation of *KCNB1* promotes adipogenesis and fat mass expansion in humans.

Recent work further demonstrated that *Kcnb1* forms a stable complex with the leptin receptor in hypothalamic neurons; loss of *Kcnb1* impairs leptin signaling and alters food intake and body composition (57). Together, these findings support a pleiotropic model in which *KCNB1* integrates metabolic signaling across adipose tissue, pancreatic islets, and the central nervous system (12)(**Figure 5**). However, the mechanisms by which rs149309426 modulates *KCNB1* expression outside adipose tissue remain unknown and warrant further investigation (58, 59).

### 3.6 Limitations of the study

Nonetheless, classical replication of rs149309426 in large European-ancestry GWAS is intrinsically underpowered due to its very low allele frequency in those populations. Under such conditions, lack of replication reflects population genetics rather than absence of biological effect. Replication strategies for ancestry-informative regulatory variants must therefore be tailored to populations in which the variant segregates at considerable frequency.

In addition, although we evaluated replication in an African-ancestry cohort from the VA Million Veteran Program, no association with BMI was observed. However, this result should be interpreted with caution, as continental African populations are not equivalent to the heterogeneous African ancestry components present in admixed Brazilian individuals, which derive from multiple subcontinental regions and distinct haplotypic backgrounds.

Importantly, replication need not be limited to statistical association alone. For rs149309426, the direction of genetic association is mirrored by allele-specific enhancer activity, transcription factor binding, and consistent effects on adipocyte differentiation and body composition across human cellular models and mouse genetics. These orthogonal lines of evidence provide mechanistic replication that complements population-based association testing and support the biological relevance of this variant despite the lack of replication in external cohorts.

### 3.7 Conclusions and perspectives

Together, our findings position *KCNB1* as a previously underrated regulator of adipogenesis and body composition, linking ion channel biology to metabolic regulation. By integrating GWAS in an admixed Brazilian population with multilayer functional genomics, we demonstrate how embedding genetic associations within their regulatory context can uncover causal non-coding variants with clear biological effects. More broadly, these results highlight the importance of ancestry-aware functional genomics for resolving the genetic architecture of complex traits such as obesity. Future studies should delineate how regulatory variation at the *KCNB1* locus shapes metabolic cross-talk among adipose tissue, pancreatic islets, and the central nervous system across diverse genetic backgrounds.

## 4 Material and methods

This study was approved by the ethics committee from Hospital das Clínicas HCFMUSP, of University of São Paulo, Brazil (CAAE: 87023418.5.0000.0068).

### 4.1 Human population study

#### 4.1.1 Study cohort, phenotyping, and genotyping

This study was conducted with data from the Baependi Heart Study, a family-based longitudinal study of genetic and environmental risk factors for cardiovascular diseases, based in a rural town in Brazil called Baependi, located in the state of Minas Gerais. The study design, recruitment process, and demographic details of this cohort have been described elsewhere (9). For this analysis, we selected data from 1,695 individuals from 95 families (**Supplementary Table 1**), randomly chosen in 2005-2006, who completed the administered questionnaire and attended a clinic visit. Briefly, probands were identified through a multi-stage random sampling strategy. Eleven of the twelve census districts were selected, followed by random selection of streets and households within each district. All individuals aged ≥18 years residing in selected households were considered eligible, and individuals younger than 18 years were excluded.

After enrollment of each proband, their first-, second-, and third-degree relatives, as well as relatives of their spouses, were invited to participate, provided they were aged ≥18 years. First-degree relatives were initially contacted by phone, including those residing in Baependi and nearby areas. Clinical evaluations were conducted in a dedicated and accessible facility within the city.

Clinical evaluations were conducted in a dedicated and accessible facility within the city, during which anthropometric and clinical measurements were evaluated for height, weight, smoking status, blood pressure, and medication use. Height and weight were measured using calibrated equipment, and body mass index (BMI) was calculated as weight (kg) divided by height squared (m²). Blood pressure was measured after 5 minutes of rest in the seated position, using the mean of three readings. Fasting blood samples (12 h) were collected to measure glucose, total cholesterol, lipoprotein fractions, and triglycerides using standardized procedures (9). BMI data were available for 1,079 individuals who attended the clinical visit and had complete anthropometric measurements.

The 1,079 DNA samples from Baependi population were genotyped for 843,039 variants using the Affymetrix 6.0 GeneChip. SNPs were imputed using the 1000 Genomes Project phase 3 (1KGP3) reference panel and the IMPUTE2 program. The 1KGP3 panel includes diverse populations, such as admixed American (AMR), African, and European groups, making it suitable for genotype imputation in admixed populations like Baependi, which reflects varying contributions from these ancestries. After imputation, 36,345,621 SNPs were available for further analysis.

#### 4.1.2 Quality control and population structure

We used human genome reference GRCh37 and applied standard quality controls after imputation using PLINK2 software (60): minor allele frequency (MAF) ≥ 1%, missing rate per variant < 10%, missing rate per individual < 10%, Hardy-Weinberg equilibrium test with p-value ≥ 1 x 10–^6^ performed in unrelated individuals to avoid bias, and imputation quality score (R^2^ > 0.8). After quality control, the Baependi dataset comprised 8,683,634 variants and 1,079 individuals, with all participants pacing the predefined QC criteria.

Principal component analysis (PCA) was used to assess population structure. SNP pruning was conducted using PLINK version 2.0 (PLINK filter –indep-pairwise 1000 50 0.05), and relatedness was assessed with kinship2 R package (version 1.6.3). We then performed PCA using PLINK2 considering only unrelated individuals (up to the 3rd degree of relatedness), and projected the resulting principal components into the related individuals.

#### 4.1.3 Body mass index genome-wide association study

The genome-wide association analysis was performed using a polygenic mixed model, in which BMI was regressed on gender, age, age^2^, and the first four principal components (PCs), with relatedness modeled as a random effect using the kinship matrix. Residuals from this model were inverse-normally transformed to obtain a standard normal distribution and subsequently used in the association analysis with each variant, assuming an additive genetic model, with no other covariates. Association analysis was performed with the R kinship2 package (version 1.6.3).

#### 4.1.4 Genomic risk loci

All SNPs that reached p-value < 1 × 10^-4^ in the association analysis were grouped into 100 kb regions. Within each region, the SNP with the lowest p-value was defined as the lead SNP for the locus. A 100 kb window was then centered on each lead SNP. If overlapping windows were generated, they were merged and considered as a single genomic locus.

### 4.2 Computational integration to identify functional evidence of obesity-associated variants

#### 4.2.1 Identification of known loci and loci selection

To determine whether a locus had been previously associated with obesity-related traits, we queried the GWAS catalog (www.ebi.ac.uk/gwas/, version 1.0.2, last updated on September 22, 2024) to identify previously reported variants located within our defined risk loci (i.e., within the 100 kb regions centered on the lead SNPs). We considered studies annotated with the following terms in “mapped traits” or “disease traits” fields in GWAS catalog database: “body mass index”, “BMI”, “waist-hip ratio”, “WHR”, “obese”, “obesity”, “weight”, “body weight”, “overweight”, “waist circumference”, “hip circumference”, “body fat”, “fat distribution”, and “fat-free mass”.

For downstream analyses, we included: (i) novel risk loci containing variants that reached genome-wide significance in our study (p-value ≤ 5 x 10^-8^), and (ii) loci containing variants that reached a lenient threshold of association (5 × 10^-8^ < p < 1 × 10^-4^) in our analysis and had also been reported in the GWAS catalog for obesity-related traits at the same lenient threshold. This approach allowed us to capture loci with consistent but weaker associations across studies, which might not reach the conventional genome-wide significance threshold.

#### 4.2.2 Selection of SNPs within regulatory regions in adipocyte cells

From 100 kb selected loci, we assessed whether the SNPs with p-value < 1 x 10–^4^ in our association analysis were in robust enhancer regions in adipose nuclei cells (E063) from Roadmap Epigenomics Consortium data considering 18-chromatin state model (5 histone modification marks and H3K27ac in 98 epigenomes), using bedtools software (61). Furthermore, we selected the most relevant SNPs that were within active enhancer considering the 18-chromatin state model, that represents regions with H3K4me1 mark together with H3K27ac and/or H3K36me3 marks in adipose nuclei (62).

#### 4.2.3 Fine-mapping with SuSiE

To prioritize candidate causal variants, we performed statistical fine-mapping using the Sum of Single Effects (SuSiE) framework (28). The analysis was conducted using GWAS summary statistics across the full locus, incorporating linkage disequilibrium (LD) structure derived from the Admixed American (AMR) population of the 1000 Genomes Project. We allowed for multiple causal variants per locus (minimum of two) and included variants with pairwise LD up to *r*2<0.99. The number of effect components (signals per locus) was set using the default parameters implemented in SuSiE. Posterior inclusion probabilities (PIPs) and credible sets were used to evaluate the likelihood of individual variants being causal.

#### 4.2.4 Selected SNPs allele frequencies in general population

We analyzed the allele’s frequencies from the selected SNPs within regulatory regions in different population from 1000 genome database Phase 3, Human Genome Diversity Project (HGDP) (63), and gnomAD v3 genomes (64). Additional population allele frequency data were obtained from dbSNP (https://www.ncbi.nlm.nih.gov/snp/docs/about/), including datasets derived from large-scale sequencing projects (e.g., TOPMed and ALFA). Allele frequencies were compared descriptively with those observed in our Brazilian cohort. Detailed list of populations and corresponding allele frequencies is provided in **Supplementary Table 7**.

### 4.3 Identification of target genes of the BMI-associated variants (HiC)

To identify possible target genes regulated by the selected enhancers and affected by BMI-associated SNPs in our study, we used *in situ* promoter capture HiC data from differentiated SGBS human preadipocytes (days 0, 2, 8 and 16 of adipocytes differentiation) generated by Sobreira et al, 2021 (12) and available in the ArrayExpress - Functional Genomics Data from EMBL-EBI repository under accession number E-MTAB-10488. Processed interaction files (ibed format) were intersected with the genomic positions of selected variants using bedtools software. Genes were assigned based on all promoter interactions overlapping SNP-containing enhancer regions. Interaction results were visualized in the WashU Epigenome Browser (https://epigenomegateway.wustl.edu/browser/). We further examined the expression patterns of genes whose promoters interacted with the selected enhancer regions in SGBS human preadipocytes during differentiation, using processed RNA-seq data also reported by Sobreira et al, 2021 (12).

### 4.4 Identification of allele-dependent enhancer activity in human preadipocyte and adipocyte cell

We conducted gene reporter assays to validate experimentally that the regions showing epigenomic signatures associated with enhancer function in adipose tissue from the Roadmap Epigenomics consortium indeed act as enhancers in human adipocytes and are modulated by variants associated with BMI in Brazilian population.

#### 4.4.1 Reporter gene assay

We assessed the activity of candidate regulatory elements containing each allele of selected variants (chr20: rs149309426 and rs141766039) by cloning them upstream of a luciferase gene containing a minimal promoter, transferring the reporter constructs into human preadipocytes and differentiated adipocytes, and measuring expression of the luciferase reporter gene to quantify the activity of the selected enhancers and assess if there was difference of enhancer activity between risk and reference allele.

We cloned 500bp-enhancer regions into pENTR-D-TOPO vector using the pENTR-D-TOPO kit (K240020, Thermo Fisher Scientific, USA), and subcloned them into pGL4.23 firefly luciferase reporter vector (E8411, Promega, USA) containing the gateway cassette, in the forward orientation, using the Gateway LR Clonase Enzyme Mix kit (11791020, Thermo Fisher Scientific, USA). For each enhancer and variant selected, we synthesized both risk and reference allele (gBlocks – IDT), centering them in the middle of the 500bp interval.

To verify the integrity of the sequences in the vectors, we used restriction enzyme assays (PvuII and EcoRV) and Sanger sequencing for both pENTR/D-TOPO and pGL4.23 vectors. To amplify and extract the vectors, we used One-Shot Top10 chemically competent E. coli (C404003, Thermo Fisher Scientific, USA) and the Wizard Plus SV Minipreps DNA Purification Kit (A1460, Promega, USA) or Plasmid Plus Maxi Kit (12965, QIAGEN, Germany).

We cultured the human preadipocyte HPrAD cells (PT-5020, Donors 32429, 27240, 27458, 27271 and 33226 - Lonza, Switzerland) following the manufacturer’s recommendations using PGM2 medium. To promote adipose differentiation of the HPrAD cells, we grew cells to confluence and induced adipocyte differentiation using preadipocytes basal medium-2 supplemented with PGM™-2BulletKit™ for 10 days, following manufacturer’s instructions (PT-8002, Lonza, Switzerland). We maintained cells at 37 °C, 5% CO2.

We co-transfected human preadipocytes and adipocytes (1 x 10^5^ cells) with 1ug of the respective pGL4.23 constructs with the pGL4.73 Renilla luciferase vector (E6911, Promega, USA) with a proportion of 1:20 (pGL4.73: pGL4.23) using Neon™ electro-transfection system (MPK5000, Thermo Fisher Scientific, USA) (3 pulses of electric shock at 1400V for 10 ms). After transfection the cells were seeded into 24 well plates for 24h for resting and then for additional 24h in 500 µL of medium III with absence or presence of LPS (1 µg/mL). All experiments were done in triplicates and we used the pGL3 vector, containing the SV40 promoter and enhancer, as a positive control, and pGL4.23 without enhancer as negative control.

Twenty-four hours after medium exchange with or without LPS, used to induce an inflammatory stimulus in adipocytes, we washed cells with PBS and lysed in Passive Lysis Buffer for 15 min under 37 °C following the Dual-Luciferase^®^ Reporter Assay System protocol (E1910, Promega, USA). We measured firefly and Renilla luciferase activity using a Sirius luminometer and FB12 Tube (Titertek Berthold, Germany). We calculated the ratios of firefly luciferase expression to Renilla luciferase expression and normalized it to the negative control vector.

#### 4.4.2 Experimental validation that rs149309426 risk allele alters transcription factor binding

To assess if the risk allelic enhancer activity and increase in *KCNB1* expression is influenced by differential transcription factor binding, we performed electrophoretic mobility shift assay (EMSA) using nuclear extracts of HeLa cells (CCL-2, ATCC, USA), a well-established source of nuclear protein for detecting DNA-protein interactions and allele-specific binding differences.

HeLa cells were routinely cultured in Dulbecco’s modified Eagle’s medium (DMEM) supplemented with 10% fetal bovine serum and maintained at 37 °C in a humidified atmosphere containing 5% CO_2_, and were used for preparation of nuclear extracts for electrophoretic mobility shift assays.

Nuclear protein extracts were prepared from HeLa cells using ProteoExtract^®^ Subcellular Proteome Extraction kit (539790, Merck, Darmstadt, Germany), recovering the supernatant and storing at -80 °C.

To test if the variant rs149309426 shows allele-specific binding to transcriptional regulators, we performed EMSA using the rs149309426 surrounding sequence (31bp) containing the two allelic forms. Double stranded oligonucleotides 3’ biotinylated probes were commercially synthesized containing either the risk or the reference allele (IDT - Iowa, USA, **Supplementary Table 10**). Binding reactions were performed using nuclear extracts, and competition assays were conducted using unlabeled oligonucleotides corresponding to the same enhancer sequences used in vector construction. Negative controls consisted of labeled probes (C or T allele) incubated without nuclear extract. As a positive control, incubation with HeLa nuclear extracts consistently produced clear shifted bands, confirming the presence of DNA–protein interactions under the assay conditions.

We conducted DNA-protein binding reactions in loading buffer, poly (dI-dC), glycerol, NP-40, MgCl_2_ and EDTA pH8, using LightShift™ Chemiluminescent EMSA kit (20148, ThermoFisher, Massachusetts, USA). For DNA-protein interaction, we incubated 10 μg nuclear extract from Hela cells with 100 fmol of labelled probes or/and with 5000 fmol of competitor probes for 40 min at room temperature followed by 6% non-denaturing polyacrylamide gel electrophoresis for 2 hours at 100V, transfer to a positively charged nylon sheet (6cmx8cm, 11209299001, Roche, Basel, Switzerland) and crosslink with UV crosslinker (Stratagene, California, USA). We detected the bands using LightShift™ Chemiluminescent EMSA kit (20148, ThermoFisher, Massachusetts, USA) and Amersham™ ImageQuant™ 800 (CYTIVA, USA). Band intensities were quantified by densitometric analysis using ImageJ. The intensity of shifted (bound) and free probe bands was measured under identical exposure conditions, and values were normalized using probe-specific no–nuclear extract controls. Quantification was performed across three independent experiments to assess allele-specific differences in DNA–protein binding.

To identify candidate transcription factors potentially interacting with the variant region, we performed in silico motif analysis using the R package *motifmatchr* (function *matchMotifs*). Genomic sequences encompassing each allele were queried against position weight matrices from the ENCODE database. This analysis was used to assess whether allelic variation altered predicted transcription factor binding sites.

#### 4.4.3 Validation of imputed rs149309426 in Brazilian sample

Since rs149309426 genotypes in the Brazilian population was obtained through imputation, we validated the genotypes using rhAmp SNP assay. The assay was custom-designed by the manufacturer based on genomic sequencing flanking rs149309426. The following primers were used - Fw: CCTTTGAGCCTTCCAACAAG and Rv: GGTAGGTGGATGGATGGATG. Genotyping was carried out with rhAmp genotyping master mix (10009823, IDT, USA), rhAmp reporter mix with reference dye and genomic DNA, following manufacturer’s recommended protocol (IDT, USA) in 384-well plates on QuantStudio 12K Flex (4471087, Applied Biosystems). To validate assay specificity and genotype calling accuracy, we included custom synthetic gBlocks DNA fragments (IDT, USA) representing both allelic states of rs149309426.

A total of 1,088 samples were successfully genotyped using the rhAmp SNP assay. Among these, genotype calls were obtained for 1,087 individuals with valid results (TT, TC, or CC), indicating a near-complete genotyping success rate.

### 4.5 *KCNB1* role in adipogenesis in human pre-adipocytes

#### 4.5.1 Human adipocyte differentiation using the *KCNB1* inhibitor, GxTx1E

To verify the effect of *KCNB1* downregulation on the adipocyte differentiation, we assessed adipocyte differentiation following the same protocol and preadipocytes cells described previously with different concentrations of Guangxitoxin-1E (0, 1, 4, 16 nmol/L; ab141648, Abcam, Cambridge, UK). After 12-days differentiation, we quantified adipocytes by Oil Red, calculating the total area with cells marked with oil red in a 5% field of 20x magnification. The experiments were done in triplicates and calculations were automated using CellProfiler (Broad Institute, Massachusetts, USA).

We also extracted RNA for qRT-PCR analysis, using RNeasy Micro Kit from Qiagen (74004, Hilden, Germany). RNAs were reverse transcribed to cDNA using Superscript IV reverse transcriptase (18090050, Thermo Fisher, Massachusetts, USA) and expression of *LPL, PPARγ* and *FASN* genes were assessed using QuantiTect SYBR Green RT-PCR Kit and QuantStudio 12K Flex (Applied Biosystems). Candidate reference genes were evaluated for expression stability using NormFinder, geNorm, BestKeeper, and the comparative ΔCt method, and the most stable gene (GAPDH) was selected for normalization. Data was presented as relative expression to preadipocytes (**Supplementary Table 10**).

### 4.6 *KCNB1* role in body adiposity in mice

#### 4.6.1 Body composition of *KCNB1* knockout mice

Fat mass, lean mass and body weight data from *Kcnb1* knockout mice was obtained from International Mouse Phenotyping Consortium (IMPC) (29, 30). We restricted the analysis to C57 control mice and *Kcnb1*−/− mice that were phenotyped on the same experimental days, ensuring that body composition measurements were obtained under comparable conditions and minimizing potential batch effects. Data are publicly available in https://www.mousephenotype.org/data/genes/MGI:96666.

### 4.7 Statistical analysis

Statistical analyses were performed using Student’s t-test (reporter gene assay, gene expression and body composition from mice model) and one-way ANOVA (EMSA quantification and adipocyte differentiation under *Kcnb1* inhibitor) to compare the means of two or multiple groups, respectively, using the graphpad prism software version 8.0 or R software version 4.5.0. P-values less than 0.05 were considered statistically significant and expressed as mean ± standard deviation.

## Data Availability

The original contributions presented in the study are included in the article/**Supplementary Material**, further inquiries can be directed to the corresponding author/s.
