# Supplementary material for: Ancestry-informative regulatory variants at KCNB1 modulate adipogenesis and body mass index
Source: Front Endocrinol (Lausanne). 2026 May 7;17:1805824. doi: 10.3389/fendo.2026.1805824 (PMC13189953; doi:10.3389/fendo.2026.1805824)
Supplement: Supplementary file 1 [file DataSheet1.docx]

Supplementary Material

Ancestry-informative regulatory variants at KCNB1 modulate adipogenesis and body mass index

# Supplementary Figures and Tables

## Supplementary Figures


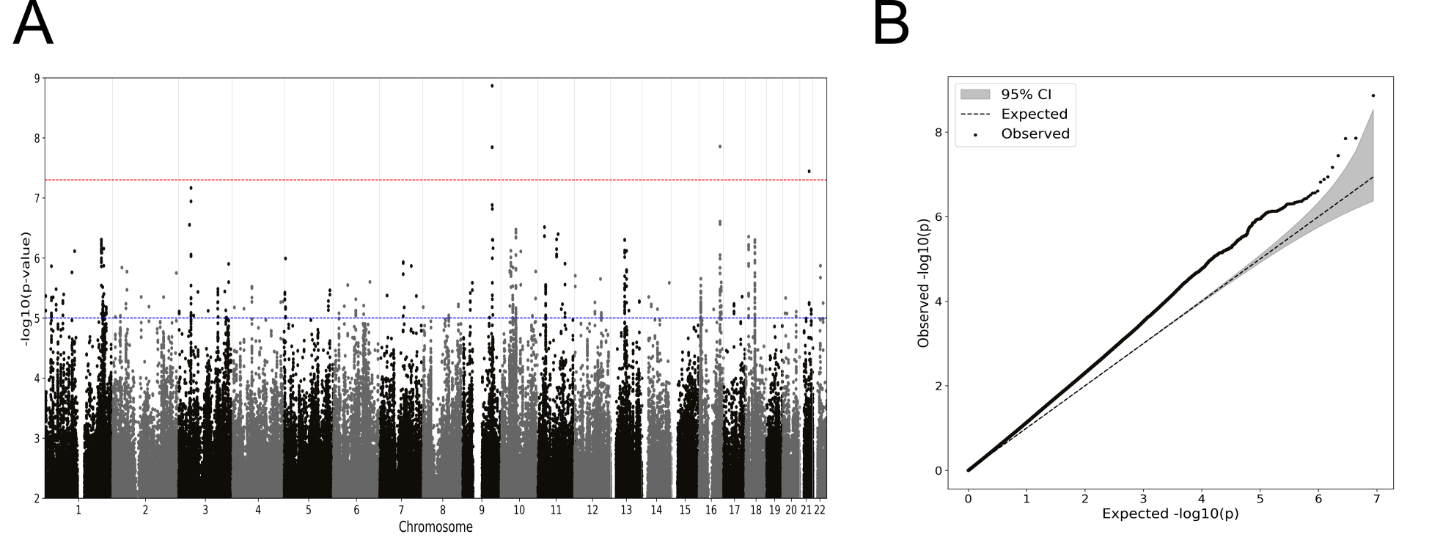


**Supplementary Figure 1.** Q–Q plot and Manhattan plot of BMI GWAS.


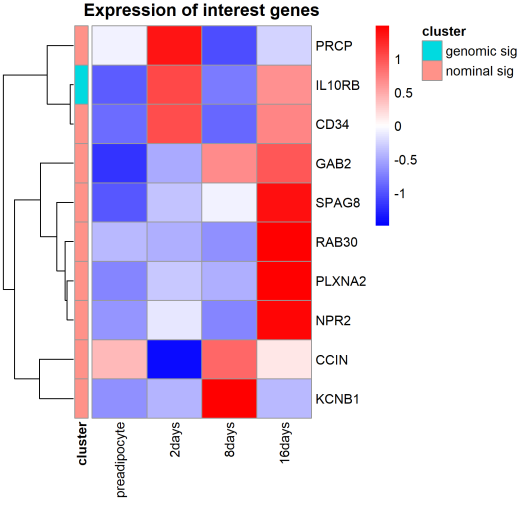


**Supplementary Figure 2.** Expression trajectory of IL10RB and KCNB1 during adipocyte differentiation.

# Supplementary Tables

**Supplementary Table 1.** Demographic and anthropometric characteristics of study participants.

| Personal Data | |
| --- | --- |
| N | 1079 |
| Age | 44.8 ± 16.7 |
| Gender, male (%) | 42.0% |
| Height (cm) | 163.9 ± 9.50 |
| BMI | 24.8 ± 4.9 |
| Clinical Analysis | |
| Diabetes (%) | 10.9 |
| Hypertension (%) | 35.5 |
| WHR (cm) | 88.1 ± 12.6 |
| SBP (mmHg) | 127.2 ± 19.7 |
| DBP (mmHg) | 79.3 ± 11.2 |
| Fasting glucose (mg/dL) | 95.5 ± 32.0 |
| Cholesterol, total (mg/dL) | 181.7 ± 48.2 |
| LDL (mg/dL) | 98.4 ± 43.4 |
| HDL (mg/dL) | 56.2 ± 16.1 |
| Triglycerides (mg/dL) | 137.3 ± 77.2 |

Data are shown as mean ± standard deviation (SD). Hypertension were considered people with SBP ≥ 140 mmHg or DBP ≥ 90 mmHg or use of anti-hypertensive drugs. Diabetic were considered people with fasting glucose ≥ 126 mg/dL or use of hypoglycemic drugs. WHR: waist-hip ratio; SBP: systolic blood pressure; DBP: diastolic blood pressure; LDL: low-density lipoprotein; HDL: high-density lipoprotein.

**Supplementary Table 10.** Oligonucleotides used in this study.

| **PCR primers** | | | |
| --- | --- | --- | --- |
| **Gene** | **Forward / Reverse** | | **Annealing (^o^C)** |
| LPL | GTGGCCCGGTTTATCAACTG | | 59.2 |
|  | TGGATCGAGGCCAGTAATTCTG | | 59.9 |
| PPARγ | TCAAGAGTACCAAAGTGCAATCA | | 58.3 |
|  | TCTCCGGAAGAAACCCTTGC | | 60.0 |
| FASN | CTACACCCAGAGCTACCGGG | | 61.4 |
|  | CACCCTGTTGTGCTCCATGT | | 60.5 |
| GAPDH | CATCCTGGGCTACACTGAGC | | 60.0 |
|  | CGTTGTCATACCAGGAAATGAGC | | 60.0 |
| **3’ biotinylated EMSA probes** | | | |
| **SNP** | **Allele** | **Sequence** | |
| rs149309426 (T) | Reference allele | CTTAATTTTTCTGTCTCTGCTACAGTGCCTA | |
| rs149309426 (C) | Alternative allele | CTTAATTTTTCTGTCCCTGCTACAGTGCCTA | |
